# Supplementary material for: The Impacts of Periconceptional Alcohol on Neonatal Ovaries and Subsequent Adult Fertility in the Rat
Source: Int J Mol Sci. 2024 Feb 20;25(5):2471. doi: 10.3390/ijms25052471 (PMC10931617; doi:10.3390/ijms25052471)
Supplement: Supplementary file 1 [file ijms-25-02471-s001.zip › ijms-2856703-supplementary.pdf]

Table S1: Details of primers used for quantitative PCR. Genes of interest are known regulators of primordial follicle growth, survival or activation/recruitment. Ten potential endogenous control genes were also tested, with only *Hprt* able to be used in analyses (all other candidates were regulated by ethanol treatment). All primer assays were Assay-on-Demand primer/prober sets (Thermo Fisher Scientific, Waltham, MA, USA).

Gene name	Gene symbol	Assay ID	Amplicon size (bp)	Accession number(s)
Genes of interest				
BCL2 associated X, apoptosis regulator	*Bax*	Rn01480161_g1	63	NM_017059.2
BCL2 antagonist/killer 1	*Bak1*	Rn00587491_m1	64	NM_053812.1<br>XM_006256101.2
BCL2 apoptosis regulator	*Bcl2*	Rn99999125_m1	104	NM_016993.1
BCL2 like 1	*Bcl2l1*	Rn00437783_m1	65	NM_001033670.1<br>XM_006235265.2
BCL2 binding component3	*Bbc3*	Rn00597992_m1	62	NM_173837.2
Anti-mullerian hormone	*Amh*	Rn00563731_g1	92	NM_037034.1
Phosphotase and tensin homolog	*Pten*	Rn00477208_m1	73	NM_0361606.1
Serine/threonine kinase 11	*Stk11*	Rn01535544_m1	59	NM001108069.1<br>XM_006240910.1
Inhibin subunit alpha	*Inha*	Rn00561423_m1	58	NM_012590.2
C-X-C motif chemokine receptor 4	*Cxcr4*	Rn00573522_s1	58	NM_022205.3
C-X-C motif chemokine ligand 12	*Cxcl12*	Rn00573260_m1	60	NM_001033882.1<br>NM_001033883.1<br>NM_022177.3
Putative endogenous control genes				
Hypoxanthine phosphoribosyltransferase 1	*Hprt1*	Rn01527840_m1	64	NM_012583.2
Actin beta	*Actb*	Rn00667869_m1	91	NM_031144.3
Ribosomal protein L13	*Rpl13*	Rn00821258_g1	68	NM_031103.1
Ribosomal protein L19	*Rpl19*	Rn07318581_g1	106	NM_031103.1
Mitochondrial ribosomal protein L1	*Mrpl1*	Rn01443873_m1	169	NM_001105997.2<br>XM_008770009.1
Phosphoglycerate kinase 1	*Pgk1*	Rn01474008_gH	142	NM_053291.3
Succinate dehydrogenase complex flavoprotein subunit A	*Sdha*	Rn00590475_m1	59	NM_130428.1
Peptidylprolyl isomerase A	*Ppia*	Rn00690933_m1	149	NM_017101.1
Eukaryotic 18S ribosomal RNA	*18S*	Hs99999901_s1[a]	187	X03205.1

[a] usable in rat samples.
